# The Causes of Chest Pain in Children and the Criteria for Targeted Myocardial Enzyme Testing in Identifying the Causes of Chest Pain in Children

**DOI:** 10.3389/fcvm.2021.582129

**Published:** 2021-03-02

**Authors:** Li Chen, Hongzhou Duan, Xiaoyan Li, Zuozhen Yang, Meng Jiao, Kangtai Sun, Mei Jin

**Affiliations:** ^1^Department of Pediatric Cardiology, Beijing Anzhen Hospital, Capital Medical University, Beijing, China; ^2^Department of Neurosurgery, Peking University First Hospital, Beijing, China; ^3^Beijing Institute of Heart, Lung and Blood Vessel Diseases, Capital Medical University, Beijing, China; ^4^Ministry of Education, Laboratory of Biosystem Homeostasis and Protection, College of Life Sciences, Zhejiang University, Hangzhou, China; ^5^Ministry of Science and Technology of the People's Republic of China, Beijing, China

**Keywords:** chest pain, children, cardiac, myocardial enzymes, diagnostic procedure

## Abstract

**Aims:** Chest pain is a common complaint at pediatric cardiology clinics and often leads to an extensive cardiac evaluation. In this study, we analyzed the causes of chest pain in Chinese children and developed diagnostic procedures and criteria for targeted myocardial enzyme testing.

**Methods and Results:** We retrospectively analyzed the clinical data of patients aged below 18 years visiting our hospital for chest pain between 2005 and 2019. Based on auxiliary exams and clinical diagnosis, we developed diagnostic procedures and criteria for targeted myocardial enzyme testing in children with chest pain. A total of 7,251 children were included in this study. The chest pain was of cardiac origin in 581 patients (8.0%). The incidence of non-cardiac chest pain was significantly higher in the preschool group and the school-age group than in the adolescent group (93.5 vs. 93.8 vs. 90.3%, *P* < 0.05). Among children with cardiac chest pain, the most common concomitant symptom was chest tightness (67.0%). Myocardial enzyme testing was performed in 5,408 patients and was abnormal in 453 patients. We developed a diagnostic procedure and criteria for targeted myocardial enzyme testing using pertinent history, physical examination, and ECG findings or UCG finding. Applying the diagnostic procedure and criteria could lead to the reduction in myocardial enzyme testing while still capturing all cardiac diagnoses.

**Conclusion:** In children, chest pain is mostly benign and rarely cardiac. During diagnosis, targeted myocardial enzyme testing based on medical history and physical examination can effectively reduce resource use.

## Introduction

Chest pain is a common complaint at pediatric clinics, accounting for ~6 per 1,000 visits at pediatric emergency room (1), as well as ~1 per 40 visits at pediatric clinics in the UK (2), To date, no large studies have been conducted to investigate this topic in Chinese children. In adults, chest pain is often caused by severe cardiovascular diseases, while in children, chest pain is mostly benign and rarely cardiac (3–11). In China, however, most parents believe that chest pain in children is cardiac and life-threatening. So most parents bring their child who has chest pain to an emergency room or see a pediatric cardiologist for comprehensive cardiac exams, which affects the child's life and schoolwork and increases the costs to their families. Therefore, it is important to investigate common causes of chest pain in children and develop diagnostic procedures in order to reduce stress to children and their families.

Collins et al. (12), Harahsheh et al. (13), and Etuwewe et al. (14) proposed evaluation methods and procedures for chest pain in children (12–14). In 2011, Friedman et al. (15) investigated the use of echocardiography (UCG) for the diagnosis of chest pain in children and developed diagnostic and treatment procedures based on medical history, physical examination, and electrocardiogram (ECG). Myocardial enzyme tests are currently widely used for the diagnosis of many heart diseases. To date, no studies have investigated targeted cardiac enzyme testing for the diagnosis of chest pain in children. It is important to develop the criteria for targeted myocardial enzyme testing in children with potential cardiac chest pain in order to reduce the economic burden on their families and reduce resource use. In this study, we analyzed the common causes of chest pain in children and the pattern of clinical visits and developed diagnostic procedures and criteria for targeted myocardial enzyme testing.

## Materials and Methods

### Case Selection

We retrospectively analyzed the clinical data of children aged 18 years or below who visited Beijing Anzhen Hospital for chest pain between January 1, 2005 and December 31, 2019. The diagnosis was coded based on the 10th revision of the International Statistical Classification of Diseases and Related Health Problems (ICD-10). Patients with incomplete clinical data or known history of heart disease were excluded from this study. We identified patients on the basis of ICD-10 billing codes for chest pain. Our study complies with the Declaration of Helsinki, and the institutional review board for clinical research at Beijing Anzhen Hospital approved the use of patient medical records for this retrospective review.

We analyzed the demographics, history of the present illness, past medical history, family history, concomitant symptoms, clinical department for initial visit, and auxiliary exams. Based on criteria that were applied in retrospect, chest pain was divided into cardiac or non-cardiac (3–7, 9–13, 15, 16) (Tables 1, 2).

**Table 1:** Cardiac causes of chest pain (3–7, 9–13, 15, 16).

**Cardiac causes**
Hypertrophic cardiomyopathy
Dilated cardiomyopathy
Coronary ischemia
Coronary artery spasm
Coronary-pulmonary arterial fistula
Coronary artery lesion caused by Kawasaki disease
Early-onset coronary artery disease due to hyperlipidemia
Cocaine abuse
Cardiac-related pulmonary hypertension
Arrhythmia
Supraventricular tachycardia,
Ventricular tachycardia
Pre-excitation syndrome
Frequent premature ventricular contractions
High-grade atrioventricular block
Inflammatory diseases
Pericarditis
Myocarditis
Suspected myocarditis
Structural abnormalities
Anomalous coronary artery origin
Large ventricular septal defect
Severe left ventricular outflow tract obstruction
Aortic valve stenosis
Mitral valve prolapse
Severe pulmonary valve stenosis
Aortic sinus aneurysm
Genetic disease
Marfan syndrome
Aortic dissection
Pericardial effusion

**Table 2:** Non-cardiac causes of chest pain.

**Non-cardiac causes**	**Included diseases**
Musculoskeletal	Costochondritis
	Precordial catch syndrome
	Muscle strain
	Trauma
	Straight-back syndrome
	Slipping rib syndrome
Respiratory	Asthma
	Pneumonia
	Bronchitis
	Pleuritis
	Acute asthmatic bronchitis
	Chronic cough
	Emphysema
	Mediastinal emphysema
	Pulmonary bullae
	Pulmonary tuberculosis
	Pleural effusion
	Pulmonary embolus
	Pneumothorax
Gastrointestinal	Gastroesophageal reflux disorder
	Esophagitis
	Gastritis
	Pancreatitis
	Esophageal hiatal hernia
	Constipation
	Gastric ulcer
	Biliary disease
Psychogenic	Conversion disorder
	Depression
	Hysteria-like attacks
	Panic/anxiety attack
Others	Skin infection
	Dysmenorrhea
	Poor wound healing
	Breast disease
	Acute chest syndrome in sickle cell disease
Idiopathic factors	

We analyzed the concomitant symptoms in cardiac and non-cardiac patients. Moreover, based on patient age at the initial visit, we divided the patients into the preschool group (3 years ≤ age ≤ 6 years), the school-age group (6 years < age ≤ 12 years), and the adolescent group (12 years < age ≤ 18 years). We analyzed the sex composition and the causes of chest pain in each group. Positive history of past illnesses included systemic lupus erythematous, juvenile rheumatoid arthritis, carnitine deficiency, and congenital adrenal insufficiency. Positive family history included sudden or unexplained death of a first-degree relative, severe familial hypercholesterolemia, pulmonary arterial hypertension, Marfan syndrome, and cardiomyopathy. Positive physical examination results included pathological heart murmur, pericardial friction rubs, abnormal second heart sounds, distant heart sounds, arrhythmia, tachycardia, bradycardia, hepatomegaly, pulsus paradoxus, edema, fever, shortness of breath, abnormal breath sounds in both lungs, and abnormal muscle strength or muscle tone.

### Interpretation of Clinical Auxiliary Exams and Clinical Diagnosis

We retrieved and analyzed ECG reports from clinical medical records. ECG abnormalities included deviation of the electrical axis, pathological ST-segment or T-wave changes, pathological Q wave, ventricular hypertrophy, atrial enlargement, supraventricular tachycardia, frequent premature ventricular contractions, pre-excitation syndrome, and high-grade atrioventricular block.

UCG, Holter test results, and chest X-ray were analyzed based on reports.

The myocardial enzyme panel included creatine kinase, creatine kinase-myocardial band, lactate dehydrogenase, cardiac troponin I, and myoglobin, which were analyzed based on laboratory reports.

We applied the criteria in retrospect to diagnose the causes of chest pain in children. The clinical diagnostic criteria of myocarditis and suspected myocarditis were listed in Table 3.

**Table 3:** The clinical diagnostic criteria of myocarditis and suspected myocarditis (16).

**Diagnostic criteria**	**Contents**
The major criteria	1. Cardiac insufficiency, cardiogenic shock, or cardiocerebral syndrome
	2. Cardiomegaly
	3. Cardiac troponin T or I (cTnT/cTnI) or creatinekinase-MB (CK-MB) increased with dynamic changes.
	4. Significant ECG changes (ECG or Holter)^#^
	5. Cardiac magnetic resonance (CMR) presents typical myocarditis features^*^
The Minor Criteria	1. History of prodromal infection, such as upper respiratory tract infection or history of gastrointestinal virus infection within 1–3 weeks before onset
	2. Chest tightness, chest pain, palpitations, fatigue, dizziness, pale, abdominal pain, etc. (at least included 2 or more of the above items); in small infants, they could have milk refusal, cyanosis, cold limbs, etc.
	3. Lactate dehydrogenase (LDH), α-hydroxybutyric dehydrogenase (α-HBDH) or aspartate transferase (AST) elevated^##^
	4. Mild abnormal ECG (refers to ST-T changes that do not meet the standard of “significant ECG changes” in the major criteria)
	5. Anti-myocardial antibody positive

# *Significant ECG changes included ST-T changes in two or more main leads (I, II, aVF, V5) with R wave as the main component lasted for more than 4 days with dynamic changes. Newly found sinus and atrioventricular block, complete right or left bundle branch block, sinus arrest, rhythmic, paired, polymorphic or multi-source premature contraction, non-atrioventricular node and atrioventricular fold ectopic tachycardia, atrial flutter, atrial fibrillation, ventricular flutter, ventricular fibrillation, QRS low voltage (except newborns), abnormal Q wave, etc*.

* *Typical myocarditis manifestations of CMR refers to at least two of the following three items: (1) myocardial edema: T2 weighted images show localized or diffuse hyperintensity; (2) myocardial congestion and capillary leakage: T1 weighted images show early gadolinium enhancement; (3) myocardial necrosis and fibrosis: T1 weighted images show at least one localized late delayed gadolinium enhancement in non-ischemic areas*.

## *In the three part of minor criteria, if serum LDH, α-HBDH or AST increased, meanwhile cTnI, cTnT or CK-MB also increased, then they are only regarded as the main indexes, and the important indexes are not calculated repeatedly*.

*Myocarditis can be diagnosed clinically if there are more than three major criteria, or two major criteria plus three minor criteria, and other diseases are excluded*.

*Suspected myocarditis can be clinically diagnosed if there are two major criteria, or 1 major criteria plus two minor criteria, or more than three minor criteria, and other diseases are excluded*.

*In the diagnostic criteria, other diseases that should be excluded include: coronary artery disease, congenital heart disease, high altitude heart disease and metabolic diseases (such as hyperthyroidism and other inherited metabolic diseases), cardiomyopathy, congenital atrioventricular block, congenital complete right or left bundle branch block, ion channel disease, orthostatic intolerance β receptor hyperfunction and ECG changes caused by drugs*.

### Diagnostic Procedures and Criteria for Targeted Myocardial Enzyme Testing for Chest Pain in Children

We developed diagnostic procedures and criteria for targeted myocardial enzyme testing in children with chest pain based on this retrospective study and the diagnostic procedures for chest pain in children from literature reports (10, 15, 17–19). In the first step of the diagnostic procedure, we should get detailed history and physical examination results. For the signs and symptoms, we should ask the patients or their parents/guardian that if they have chest tightness, fever, cough, stomach ache, abnormal mental state or palpitations associated with chest pain, syncope during activity, chest pain during exercise, history of cardiac surgery or interventions, complex congenital heart defects, Marfan syndrome, children with on specific cardiac drugs heart transplant, history of Kawasaki disease, history of drug abuse, significant family history of arrhythmias, sudden death in young adulthood, genetic disorders linked with arrhythmias e.g., Long QT syndromes or Brugada syndrome, first-degree relatives have familial hypercholesterolemia, cardiomyopathy and pulmonary hypertension. About physical examination, we should know if they have fever, abnormal breath sounds, pathologic murmur, abnormal heart sounds, hypoxia, peripheral edema, hepatomegaly, significant tachycardia or irregular rhythm. In the second step of the diagnostic procedure, if the patients have chest pain with the following history of the present illness, past medical history, family history and physical examination (History of present illness include post-exercise syncope, chest pain following strenuous exercise or accompanied by chest tightness; past medical history include cardiac surgery or intervention, complex congenital heart disease, Marfan syndrome, Kawasaki disease, drug abuse, and connective tissue disease; family history include hereditary arrhythmia, pulmonary arterial hypertension, and cardiomyopathy, sudden or unexplained death of first-degree relatives under 35 years old, and familial hypercholesterolemia among first-degree relatives; physical examination include pathological heart murmur, abnormal heart sounds, tachycardia, arrhythmia, transcutaneous oxygen saturation, peripheral edema, and hepatomegaly), they should be referred to pediatric cardiologist for diagnosis and treatment. And other patients will be assigned to different departments for diagnosis and treatment. In the third step, doctors from different department will carry out detailed physical examination and necessary auxiliary examination for patients. Based on the results of auxiliary examinations, the doctors will make diagnosis and give treatment for patients. In the diagnosis of cardiogenic chest pain, we use diagnostic criteria of myocarditis and suspected myocarditis from literature reports (16) and our clinical analysis to make the criteria for targeted myocardial enzyme testing for chest pain in children.

### Statistical Analysis

SPSS20.0 was used for statistical analysis. Measurement data are presented as mean ± standard deviation ($\bar{x}\pm$S), and count data are expressed as frequency (percentage). The χ^2^ test was performed to analyze sex distribution and the causes of chest pain. *P* < 0.05 was considered statistically significant.

## Results

### Demographics

A total of 7,251 children were included in this study, including 4,293 boys (59.2%) and 2,958 girls (40.8%), aged 3–18 years (mean: 12.1 ± 4.0). Chest pain was more common in school-age children and adolescents. Most patients in the preschool group were girls (55.5%) while most patients in the school-age and adolescent groups were boys (60.6%, 61.3%), and this difference in sex distribution was significant (*P* < 0.01).

### Pattern of Clinical Visits and Clinical Department for Initial Visit in Children With Chest Pain

**Figure 1:**
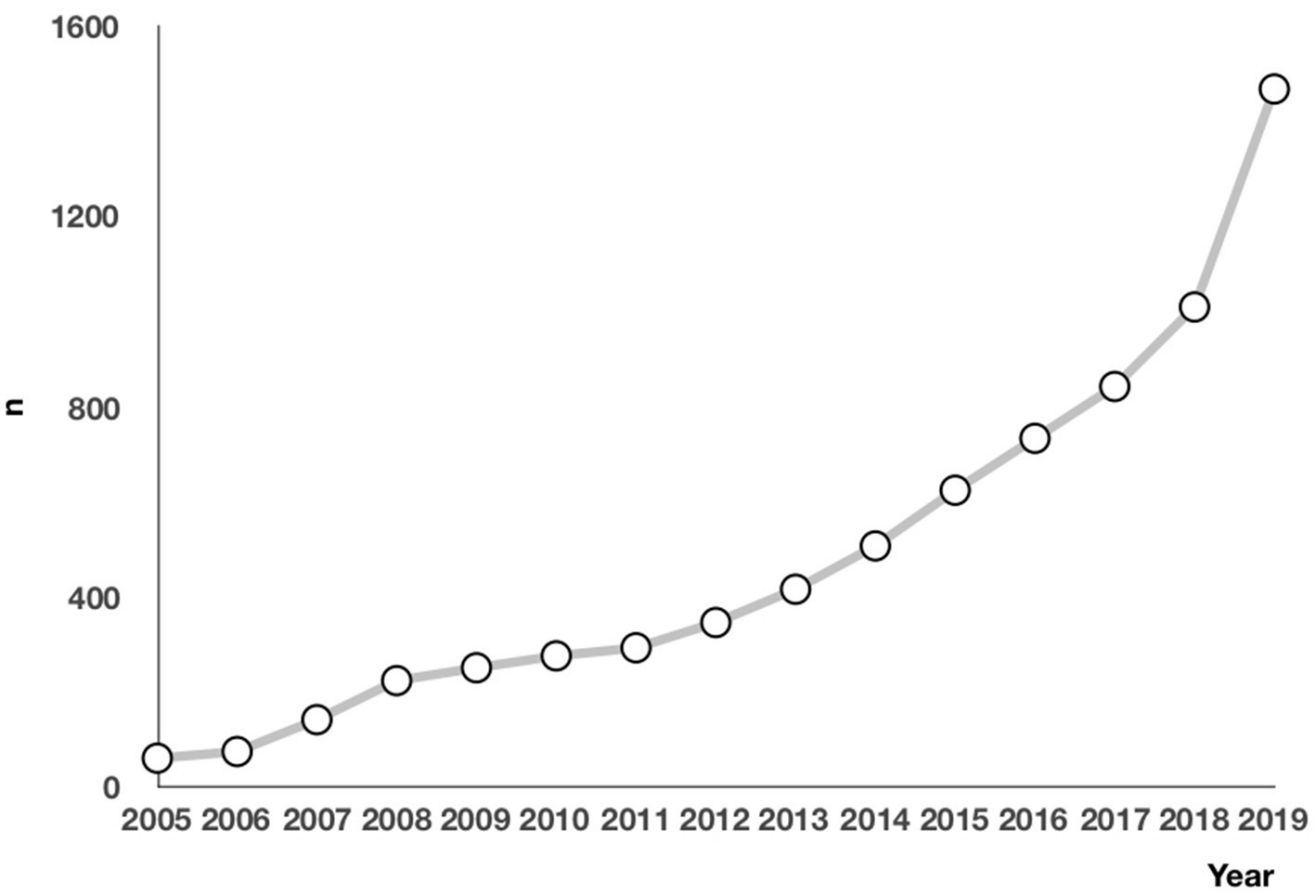
Number of initial evaluations of chest pain in children per year.

**Figure 2:**
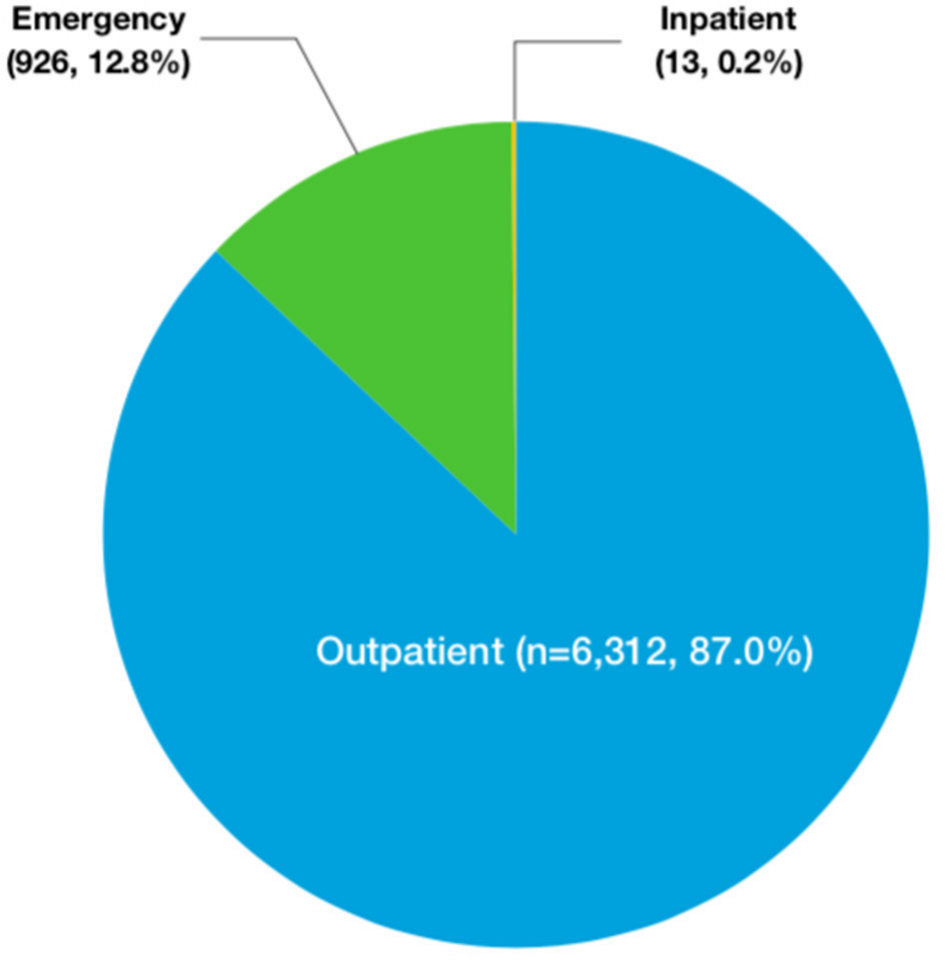
The emergency-department visits of chest pain in children.

**Figure 3:**
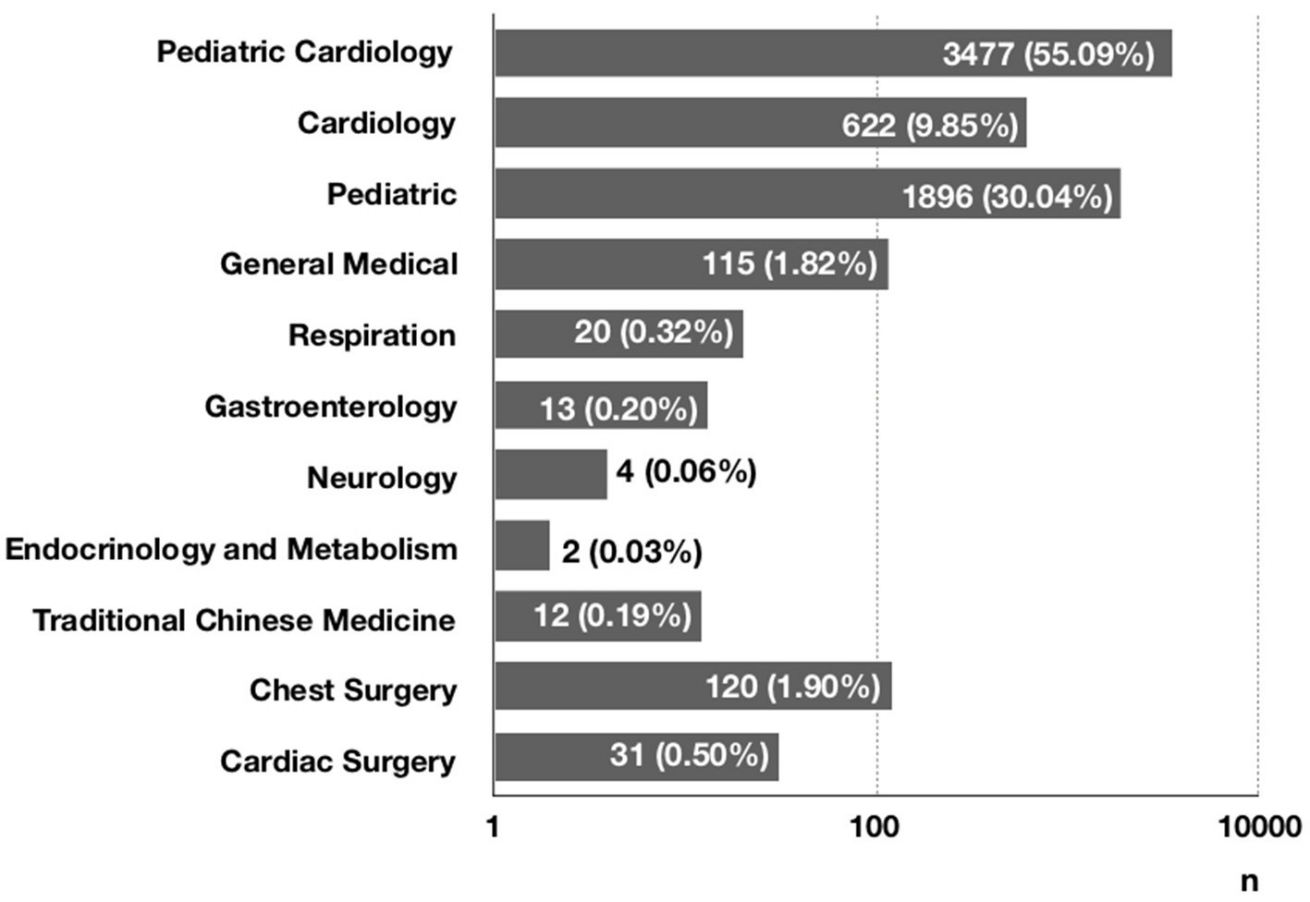
The initial visited departments of chest pain in children.

The number of children who saw a doctor for chest pain was increasing each year from 2005 to 2019 (Figure 1). Specifically, 6,312 patients (87.0%) visited a clinic, and 926 patients (12.8%) went to an emergency room. Only 13 patients (0.2%) were admitted to the hospital (Figure 2). At the initial visit, most children saw a pediatric cardiologist (*n* = 3,477, 55.09%) or a general pediatrician (*n* = 1,896, 30.04%) (Figure 3).

### The History of Past Illness, Family History, Physical Examination, and Concomitant Symptoms in Children With Chest Pain

None of the children had positive history of past illness. Four patients had a positive family history, including two patients whose fathers had dilated cardiomyopathy, one patient whose sibling had Marfan syndrome, and one patient whose father had died suddenly. Physical examination was positive in 795 children. Among them, 75 patients had pathological heart murmur, including systolic murmur (*n* = 25), diastolic murmur (*n* = 32), accentuated P2 (*n* = 3), splitting of the second heart sound (*n* = 12), and continuous machine-like murmur (*n* = 3); 33 had arrhythmia; 62 had a tachycardia; 77 had a bradycardia; 198 patients had fever and bibasilar crackles in both lungs; six had fever and wheezing sounds in both lungs; 327 only had fever; 15 only had wheezing sounds in both lungs; two had hepatomegaly.

Among children with cardiac chest pain (*n* = 581, 8.0%), the most common concomitant symptom was chest tightness (*n* = 389, 67.0%). Among patients with non-cardiac chest pain (*n* = 6,670, 72.0%), most (*n* = 4,899, 73.4%) had no concomitant symptoms (Table 4).

**Table 4:** The associated symptoms of chest pain in children.

**Associated symptoms**	***n* (%)**
Cardiac-related chest pain	581
No associated symptoms	116 (20.0%)
Chest oppression	389 (67.0%)
Palpitation	43 (7.4%)
Fatigue	32 (5.5%)
Syncope	5 (0.9%)
Dizzy	4 (0.7%)
Headache	2 (0.3%)
Fever	4 (0.7%)
Respiratory symptoms	11 (1.9%)
Gastrointestinal symptoms	5 (0.9%)
Non-cardiac related chest pain	6,670
No associated symptoms	4,899 (73.4%)
Chest oppression	666 (10.0%)
Palpitation	102 (1.5%)
Fatigue	35 (0.5%)
Syncope	14 (0.2%)
Dizzy	46 (0.7%)
Headache	11 (0.2%)
Joint pain	2 (0.03%)
Fever	238 (3.6%)
Respiratory symptoms	562 (8.4%)
Gastrointestinal symptoms	103 (1.5%)

### The Results of ECG, UCG, Holter Test, and Chest X-Ray

All 7,251 patients (100%) underwent ECG, 5,352 (73.8%) underwent UCG, 128 (1.8%) underwent Holter test, 1,037 (14.1%) underwent chest X-ray, and 5,408 (74.6%) underwent myocardial enzyme testing.

ECG was abnormal in 574 of 7,251 children (7.9%). The most common abnormality was pathological ST-segment or T-wave changes (*n* = 537, 93.6%), followed by frequent premature ventricular contractions (*n* = 7, 1.2%), pre-excitation syndrome (*n* = 10, 1.8%), supraventricular tachycardia (*n* = 7, 1.2%), deviation of the electrical axis (*n* = 6, 1.0%), pathological Q wave (*n* = 6, 1.0%), and left atrial enlargement (*n* = 1, 0.2%).

UCG was abnormal in 331 of 5,352 children (6.2%). The abnormality was unrelated to chest pain in 221 of these patients (66.8%) and was related to chest pain in 110 of them (33.2%), including aortic sinus enlargement with aortic sinus aneurysm (*n* = 3, 2.7%), coronary artery dilation (*n* = 3, 2.7%), generalized cardiomegaly with reduced left ventricular systolic function (*n* = 4, 3.6%), pericardial effusion (*n* = 2, 1.8%), large ventricular septal defect (*n* = 2, 1.8%), severe pulmonary valve stenosis with pulmonary valve regurgitation (*n* = 1, 0.9%), quadricuspid aortic valve with aortic sinus aneurysm (*n* = 1, 0.9%), aortic valve stenosis (*n* = 3, 2.7%), coronary-pulmonary arterial fistula (*n* = 4, 3.6%), anomalous origin of the left coronary artery from the pulmonary artery (*n* = 6, 5.5%), anomalous origin of the right coronary artery from the left sinus (*n* = 3, 2.7%), pulmonary arterial hypertension (*n* = 6, 5.6%), and left ventricular enlargement with normal left ventricular systolic function (*n* = 72, 65.5%).

The Holter test was abnormal in only six of 128 children (4.7%), all of whom had frequent premature ventricular contractions. Chest X-ray was abnormal in 643 of 1,037 children (62.0%). The most common abnormality was patchy opacities in both lungs (*n* = 225, 35.0%), followed by increased lung markings (*n* = 368, 57.2%), pneumothorax (*n* = 39, 6.1%), and pleural effusion (*n* = 11, 1.7%).

Myocardial enzyme tests were abnormal in 453 of 5,408 children (8.4%). In these 453 patients, 362 (80.0%) had abnormal ECG with normal UCG, 79 (17.4%) had abnormal ECG and UCG (60 had cardiac dilatation and EF normal or mild decrease, 19 had other cardiac disease), 12 (2.6%) had abnormal UCG (cardiac dilatation and EF normal or mild decrease) with normal ECG.

### Diagnostic Results of Chest Pain in Children

**Figure 4:**
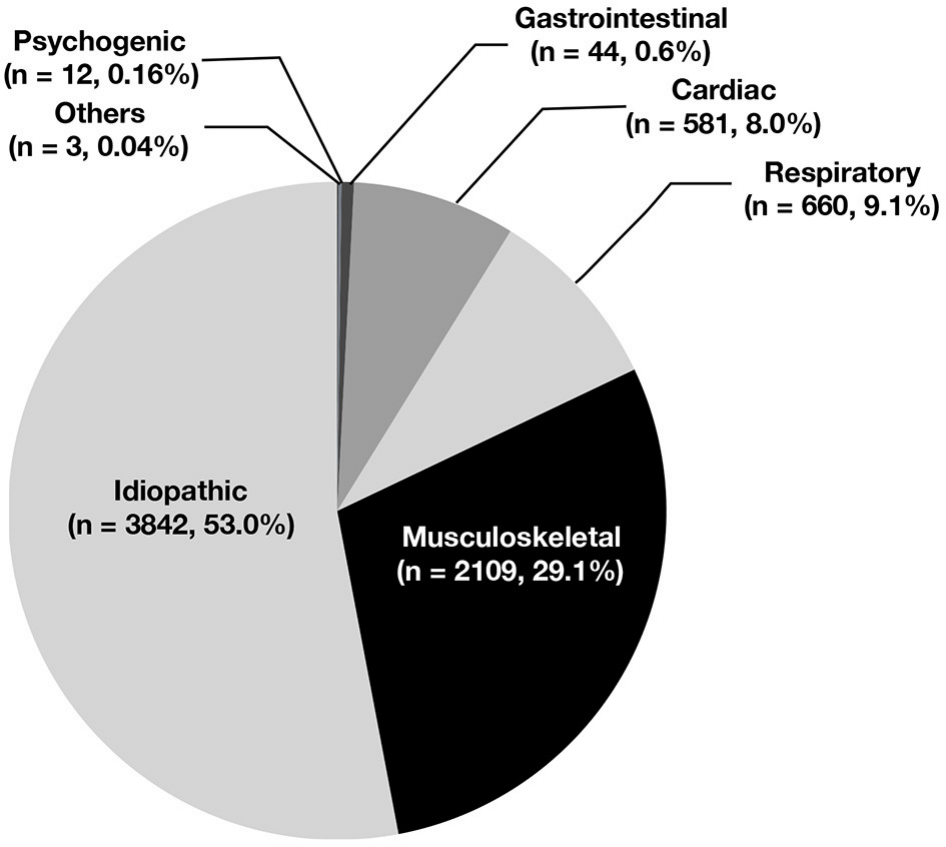
The diagnosis of chest pain in children.

Based on medical history, physical examination, and auxiliary exams, 581 patients (8.0%) were diagnosed with cardiac chest pain, and 6,670 patients (92.0%) were diagnosed with non-cardiac chest pain. Some 3,842 cases (53.0%) were idiopathic, 2,109 (29.1%) were related to skeletomuscular diseases, 660 (9.1%) were related to respiratory diseases, 44 (0.6%) were related to gastrointestinal diseases, 12 (0.16%) were related to mental diseases, and 3 (0.04%) were related to other conditions (Figure 4). The incidence of non-cardiac chest pain was significantly higher in the preschool group and the school-age group than in the adolescent group (93.5 vs. 93.8 vs. 90.3%, *P* < 0.05) (Table 5).

**Table 5:** The causes of chest pain in children with different ages.

	***n***	**3 years ≤ age ≤ 6 years**	**6 years < age ≤ 12 years**	**12 years < age ≤ 18 years**
Total	7,251	784	2,779	3,688
Cardiac	581 (8.0%)	51 (6.5%)	171 (6.2%)	359 (9.7%)
Non-cardiac	6,670 (92.0%)	733 (93.5%)	2608 (93.8%)	3,329 (90.3%)
X2		8.096	27.011	
P		0.004^#^	0.000^#^	

# *stands for P < 0.05 contrast to 12 years < age ≤ 18 years group*.

**Figure 5:**
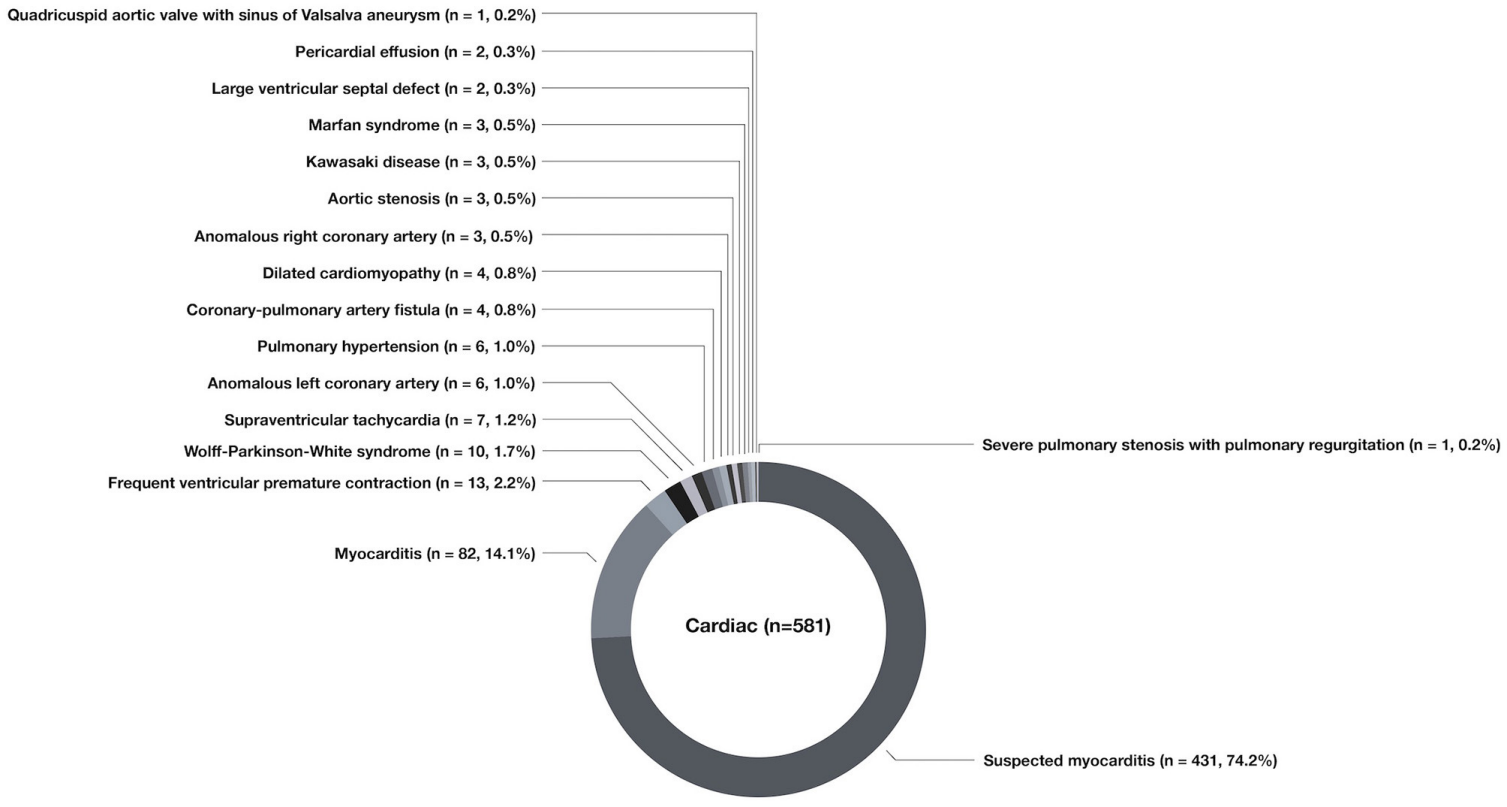
The cardiac diseases causing chest pain in children.

Among the 581 cases of cardiac chest pain, the most common cause was suspected myocarditis (*n* = 431, 74.2%), followed by myocarditis (*n* = 82, 14.1%), frequent premature ventricular contractions (*n* = 13, 2.2%), pre-excitation syndrome (*n* = 10, 1.7%), supraventricular tachycardia (*n* = 7, 1.2%), anomalous origin of the left coronary artery (*n* = 6, 1.0%), pulmonary hypertension (*n* = 6, 1.0%), dilated cardiomyopathy (*n* = 4, 0.8%), coronary-pulmonary arterial fistula (*n* = 4, 0.8%), anomalous origin of the right coronary artery (*n* = 3, 0.5%), aortic valve stenosis (*n* = 3, 0.5%), Marfan syndrome (*n* = 3, 0.5%), Kawasaki disease (*n* = 3, 0.5%), large ventricular septal defect (*n* = 2, 0.3%), pericardial effusion (*n* = 2, 0.3%), severe pulmonary valve stenosis with pulmonary valve regurgitation (*n* = 1, 0.2%), and quadricuspid aortic valve with aortic sinus aneurysm (*n* = 1, 0.2%) (Figure 5).

Six hundred and sixty cases of chest pain were caused by respiratory diseases (Table 6), and 44 cases of chest pain were caused by gastrointestinal diseases (Table 7).

**Table 6:** The respiratory diseases causing chest pain in children.

**Respiratory diseases (*n =* 660)**	***n* (%)**
Bronchitis	357 (54.1%)
Pneumonia	224 (33.9%)
Pneumothorax	38 (5.7%)
Asthma	15 (2.2%)
Pleuritis	10 (1.5%)
Acute asthmatic bronchitis	6 (0.9%)
Chronic cough	5 (0.7%)
Emphysema	1 (0.2%)
Mediastinal emphysema	1 (0.2%)
Pulmonary bullae	1 (0.2%)
Pulmonary tuberculosis	1 (0.2%)
Pleural effusion	1 (0.2%)

**Table 7:** The gastrointestinal diseases causing chest pain in children.

**Gastrointestinal diseases (*n =* 44)**	***n* (%)**
Gastritis	28 (63.6%)
Gastroesophageal reflux	13 (29.5%)
Esophagitis	1 (2.3%)
Esophageal hiatal hernia	1 (2.3%)
Constipation	1 (2.3%)

Among the 2,109 cases of chest pain due to skeletomuscular diseases, the most common cause was precordial catch syndrome (*n* = 1,308, 62.1%), followed by slipping rib syndrome (*n* = 787, 37.3%), costochondritis (*n* = 7, 0.3%), trauma (*n* = 5, 0.2%), and straight-back syndrome (*n* = 2, 0.1%). Among the 12 cases of chest pain due to mental diseases, the causes included anxiety (*n* = 5), depression (*n* = 4), and hysteria-like attacks (*n* = 3). Meanwhile, patients with mental diseases-related chest pain were all adolescents. Among the three cases of chest pain due to other disorders, the causes included breast mass (*n* = 1), dysmenorrhea (*n* = 1), and poor wound healing (*n* = 1).

### Diagnostic Procedures and Criteria for Targeted Myocardial Enzyme Testing for Chest Pain in Children

**Figure 6:**
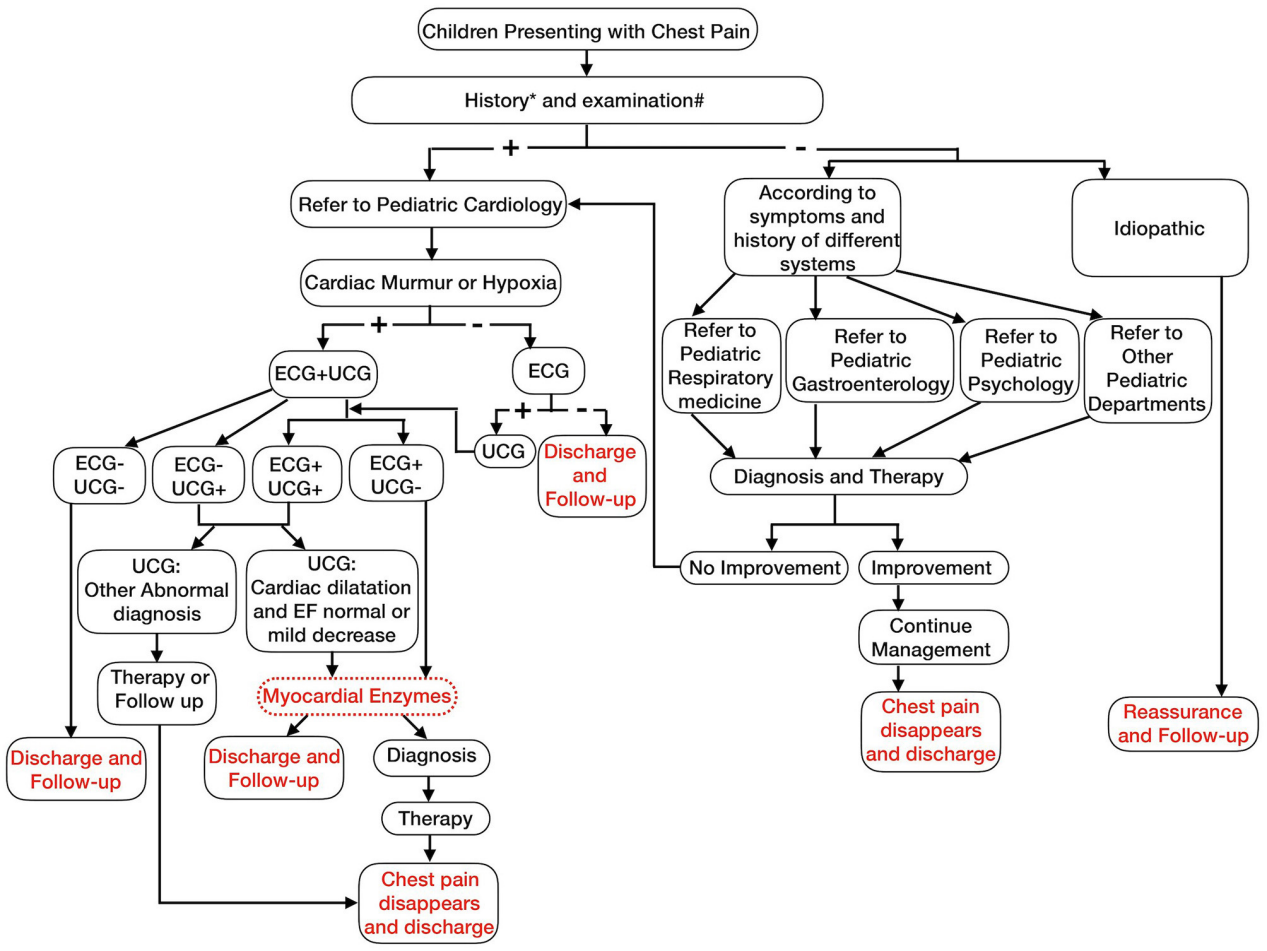
Chest pain management algorithm (History* includes fever, cough, stomach ache associated with chest pain, abnormal mental state, palpitations associated with chest pains, syncope during activity, chest pain during exercise, history of cardiac surgery or interventions, complex congenital heart defects, Marfan syndrome, children with on specific cardiac drugs heart transplant, history of Kawasaki disease, history of drug abuse, significant family history of arrhythmias, sudden death in young adulthood, genetic disorders linked with arrhythmias e.g., Long QT syndromes or Brugada syndrome, first-degree relatives have familial hypercholesterolemia, cardiomyopathy and pulmonary hypertension; Examination^#^ include fever, abnormal breath sounds, pathologic murmur, abnormal heart sounds, hypoxia, peripheral edema, hepatomegaly, significant tachycardia, or irregular rhythm).

Based on the diagnostic procedures for chest pain in children from literature reports and our analysis, we recommend that for children with chest pain, clinicians collect detailed information about the history of the present illness, past medical history, and family history and perform a comprehensive physical examination to distinguish cardiac from non-cardiac chest pain. Patients with potential cardiac chest pain should be referred to a pediatric cardiologist and undergo ECG or ECG plus UCG if they have a pathological heart murmur or hypoxemia, respectively. Patients should undergo myocardial enzyme testing if they have abnormal ECG plus normal UCG or abnormal/normal ECG plus abnormal UCG, suggesting cardiomegaly with normal or mildly reduced left ventricular systolic function. Myocardial enzyme testing is not recommended for patients with normal ECG plus normal UCG or normal ECG plus abnormal UCG, suggesting other cardiac malformations (Figure 6).

Children with non-cardiac chest pain should see an appropriate specialist based on medical history and physical examination. Patients may continue to receive treatment or follow up if the chest pain alleviates or resolves after treatment, or they can visit a pediatric cardiologist for evaluation if the chest pain does not improve after treatment (Figure 6).

For these children with chest pain, if they were evaluated by this flow chart, 580 patients will be referred to a pediatric cardiologist, and then 75 will undergo ECG and UCG examination, 505 will undergo ECG examination only. Through analyzing these 580 patients' ECG or UCG, 512 will undergo myocardial enzyme testing. Based on the diagnostic procedures, 509 patients could be diagnosed as cardiac chest pain, 72 patients with cardiac chest pain would be missed. So, the positive diagnostic value for cardiac chest pain of this flow chart was 87.6%. Meanwhile, through the diagnostic procedures, pediatric cardiac specialist outpatient visit rate and unnecessary auxiliary examination significantly reduced, respectively.

## Discussion

Chest pain is a common symptom. With the improvement of living standards and attention to quality of life, more children are seeing a doctor for chest pain. In 2011, Saleeb et al. (19) analyzed the clinical data of children who visited Boston Children's Hospital for chest pain between 2000 and 2009 and found that the number of children who saw a doctor for chest pain increased each year. This study showed a similar trend at our hospital between 2005 and 2019, suggesting that parents are paying more attention to their children's symptoms and that many parents believe that chest pain in children is cardiac and life-threatening, due to a lack of knowledge in the common causes of chest pain in children. As a result, they often take their children to an emergency or a pediatric cardiologist. In fact, most cases of chest pain in children are non-cardiac and benign (3–7, 9–11, 15, 18, 19). In 2011, Saleeb et al. (19) retrospectively analyzed the pattern of visits, causes, and mortality of chest pain in 3,700 children and found that 18% of the patients visited an emergency room and that 99% of the cases were non-cardiac. During the 4.4-years follow-up, only three patients died of non-cardiac diseases. This indicates that mortality is low in children with chest pain, with few cardiac deaths. In 2020, Gesuete et al. analyzed the clinical data of 761 children who went to an emergency for chest pain and found that only 1% of the cases were cardiac (11). Our study showed that 92.0% of the cases were non-cardiac, 12.8% of the patients went to an emergency, and 55.09% saw a pediatric cardiologist, suggesting that most parents believe that their children's chest pain is critical and has a cardiac cause. Therefore, it is important to educate the public about chest pain in children, investigate the causes of chest pain in children, and develop standard diagnostic procedures, in order to reduce resource use.

Chest pain in children may be cardiac or non-cardiac (5, 16). The causes of cardiac chest pain include ① coronary artery diseases; ② arrhythmia; ③ inflammatory diseases; ④ structural abnormalities (5, 16). The causes of non-cardiac chest pain include respiratory diseases, gastrointestinal diseases, skeletomuscular diseases, mental diseases, and idiopathic factors. In 2011, Saleeb et al. retrospectively analyzed the causes of chest pain in 3,700 children and found that 52% of the cases were idiopathic, 36% were related to skeletomuscular diseases, 7% were related to gastrointestinal diseases, 1% were related to mental disorder, and only 1% were cardiac. The most common cause of cardiac chest pain was inflammatory disease, such as pericarditis and myocarditis (19).

This is the first large study to investigate the causes of chest pain in Chinese children. We analyzed the causes of chest pain in 7,251 children who visited our hospital during a 15-years period and found that 53.0% of the cases were idiopathic, 29.1% were related to skeletomuscular diseases, 9.1% were related to respiratory diseases, 8.0% were cardiac, 0.6% were related to gastrointestinal diseases, 0.16% were related to mental diseases, and 0.04% were related to other conditions. These data indicate that idiopathic chest pain was the most common cause of chest pain in Chinese children, which is consistent with foreign reports. In our study, we also found that skeletomuscular-related chest pain was the second reason. Idiopathic and skeletomuscular-related chest pain always were not life threatening, so for most children with chest pain, their parents or guardian could not be scared and go to emergency immediately. Meanwhile, 0.16% were related to mental diseases in our study. Through analyzing their ages, we found that they were all adolescents. In adolescence, they might have study pressure, social pressure and the influence of puberty hormone level, for releasing the pressure, they complained chest pain. So for adolescents with chest pain, the parents or guardian should give them more care, and this can relieve their chest pain.

At their initial visit, most patients with chest pain see a pediatric cardiologist. Moreover, parents often request cardiac auxiliary exams to exclude cardiac chest pain. As a result, most children with chest pain undergo various unnecessary auxiliary exams. Therefore, it is important to develop diagnostic procedures for chest pain in children in order to reduce resource use. Collins et al. (12), Harahsheh et al. (13), and Etuwewe et al. (14) proposed evaluation methods and procedures for chest pain in children (12–14). The researchers stressed the importance of detailed medical history and physical examination to determine the potential cause of chest pain, followed by necessary exams. The researchers, however, did not describe how to choose the auxiliary exams. In 2011, Friedman et al. (15) reported that they developed standard clinical evaluation and treatment procedures for chest pain in children and applied these procedures in children who saw a pediatric cardiologist at Boston Children's Hospital for chest pain between 2000 and 2009. The researchers believed that patients with exercise-related chest pain or positive family history should undergo comprehensive cardiac evaluation, including UCG, for further diagnosis. With these procedures, UCG usage was reduced by 20% without missing any case of cardiac chest pain, thereby reducing resource use and the costs to the families.

Myocardial enzyme testing is widely used in clinical practice to help diagnose heart conditions. In China, many doctors order myocardial enzyme tests at the initial visit to exclude myocarditis or suspected myocarditis in children with chest pain. However, the test result is normal in most cases. In 2020, the Subspecialty Group of Cardiology, Society of Pediatrics, Chinese Medical Association, issued recommendations for the diagnosis of myocarditis in children and clearly stated the diagnostic criteria for myocarditis and suspected myocarditis in children (16). No past studies have investigated how to choose the myocardial enzyme test and reduce their use without missing any case of cardiac chest pain in children. The present study showed that for children with cardiac chest pain, the most common concomitant symptom was chest tightness (67.0%) while most patients with non-cardiac chest pain had no concomitant symptoms, and respiratory or gastrointestinal symptoms were the main concomitant symptoms (if any). Therefore, concomitant symptoms may help evaluate the potential cause of chest pain in children, followed by auxiliary exams. In this study, among the 7,251 children with chest pain, myocardial enzyme tests were abnormal in only 453 of 5,408 patients (8.4%). In these 453 patients, 362 (80.0%) had abnormal ECG with normal UCG, 79 (17.4%) had abnormal ECG and UCG (60 had cardiac dilatation and EF normal or mild decrease, 19 had other cardiac disease), 12 (2.6%) had abnormal UCG (cardiac dilatation and EF normal or mild decrease) with normal ECG. These results suggested that myocardial enzyme testing is unnecessary in patients without symptoms or signs of myocarditis or suspected myocarditis during the initial evaluation. Such an approach would help reduce resource use and unnecessary treatment.

This is the first study to develop diagnostic procedures and criteria for targeted myocardial enzyme testing in children with chest pain. Through analyzing the clinical data of 7,251 children who visited our hospital for chest pain during a 15-years period and referencing the diagnostic procedures from literature reports, we recommend that clinicians evaluate the potential cause of chest pain based on the history of the present illness, past medical history, family history, and physical examination and refer patients to an appropriate specialist. Patients with potential cardiac chest pain should undergo ECG or ECG plus UCG if they have a pathological heart murmur or hypoxemia, respectively. Patients should undergo myocardial enzyme testing if they have abnormal ECG plus normal UCG or normal/abnormal ECG plus abnormal UCG, suggesting cardiomegaly with normal or mildly reduced left ventricular systolic function. Patients with non-cardiac chest pain may continue to receive treatment or follow-up if the chest pain alleviates or resolves after treatment, or they should be referred to a pediatric cardiologist for evaluation to exclude heart conditions if the chest pain does not improve after treatment. Patients with idiopathic chest pain may receive counseling and follow-up. These procedures reduce visits and unnecessary myocardial enzyme tests, thereby reducing resource use.

There were some limitations in our study. Firstly, this was a retrospective study in one single center, so the relevant data were affected by the hospital size, first line specialty, patients' source and other aspects. The relevant results may not perfectly represent all the children patients in China. However, as one of the largest cardiovascular hospitals in China and even in Asia, our study involved the largest number of children with chest pain in China and the screening for children's heart might also be the most comprehensive, our results showed that there was no clear correlation between chest pain and cardiovascular disease in children, so we believe this result should be correct and reliable. Secondly, since this retrospective study spans 15 years, the diagnostic criteria and ICD codes of some diseases have changed during this period, which might have some impact on our research results. In order to avoid this kind of influence, we verified the diagnosis not only by diagnostic coding but also by analyzing the results of auxiliary exams. Furthermore, as the diagnostic procedures we proposed here were designed based on the results of retrospective study, prospective studies in future should be conducted to further confirm the efficacy, feasibility and superiority of the diagnostic procedure. In the future, we will conduct a large multicenter clinical study to further investigate the cause of chest pain in Chinese children, and explore a non-invasive and effective diagnostic method for children with chest pain. Meanwhile, we will conduct a prospective study to investigate the efficacy of our diagnostic procedures and criteria for targeted myocardial enzyme testing in children with chest pain. Based on the above, it can be predicted that an economic and effective diagnostic procedure for chest pain in Chinese children will be established.

## Data Availability Statement

The original contributions presented in the study are included in the article/supplementary material, further inquiries can be directed to the corresponding author/s.

## Ethics Statement

The studies involving human participants were reviewed and approved by The Ethics Committee of Beijing Anzhen Hospital, Capital Medical University. Written informed consent to participate in this study was provided by the participants' legal guardian/next of kin.

## Author's Note

Chest pain is a common complaint at paediatric clinics, accounting for ~6 per 1000 visits at paediatric emergency room. Most parents bring their child who has chest pain to an emergency room or see a paediatric cardiologist for comprehensive cardiac exams, which affects the child's life and schoolwork and increases the costs to their families. To date, no large studies have been conducted to investigate the causes of chest pain in Chinese children and no studies have investigated targeted cardiac enzyme testing for the diagnosis of chest pain in children. Our study shows that chest pain is more common in school-age children and adolescents. For Chinese children with chest pain, the most common cause is idiopathic chest pain. Few cases are cardiac chest pain. Patients with potential cardiac chest pain should undergo ECG or ECG plus UCG if they have a pathological heart murmur or hypoxemia, respectively. Patients should undergo myocardial enzyme testing if they have abnormal ECG plus normal UCG or abnormal ECG plus abnormal UCG (UCG suggests cardiomegaly with normal or mildly reduced left ventricular systolic function). This approach would reduce resource use and the costs to the families.

## Author Contributions

LC, HD, XL, ZY, MJ, KS, and MJ made substantial contributions to the conception or design of the work or the acquisition, analysis or interpretation of data. LC drafted the work. HD and MJ revised the work critically for important intellectual content. All authors read and approved the final manuscript.

## Conflict of Interest

The authors declare that the research was conducted in the absence of any commercial or financial relationships that could be construed as a potential conflict of interest.
